# Wildlife Trade and Human Health in Lao PDR: An Assessment of the Zoonotic Disease Risk in Markets

**DOI:** 10.1371/journal.pone.0150666

**Published:** 2016-03-23

**Authors:** Zoe F. Greatorex, Sarah H. Olson, Sinpakone Singhalath, Soubanh Silithammavong, Kongsy Khammavong, Amanda E. Fine, Wendy Weisman, Bounlom Douangngeun, Watthana Theppangna, Lucy Keatts, Martin Gilbert, William B. Karesh, Troy Hansel, Susan Zimicki, Kathleen O’Rourke, Damien O. Joly, Jonna A. K. Mazet

**Affiliations:** 1 Wildlife Conservation Society, Wildlife Health & Health Policy Program, Bronx, New York, United States of America; 2 University of Wisconsin Madison, Center for Sustainability and the Global Environment, Madison, Wisconsin, United States of America; 3 National Animal Health Laboratory, Department of Livestock and Fisheries, Vientiane, Lao PDR; 4 FHI360, Washington, District of Colombia, United States of America; 5 One Health Institute, School of Veterinary Medicine, University of California, Davis, California, United States of America; US Geological Survey, UNITED STATES

## Abstract

Although the majority of emerging infectious diseases can be linked to wildlife sources, most pathogen spillover events to people could likely be avoided if transmission was better understood and practices adjusted to mitigate risk. Wildlife trade can facilitate zoonotic disease transmission and represents a threat to human health and economies in Asia, highlighted by the 2003 SARS coronavirus outbreak, where a Chinese wildlife market facilitated pathogen transmission. Additionally, wildlife trade poses a serious threat to biodiversity. Therefore, the combined impacts of Asian wildlife trade, sometimes termed bush meat trade, on public health and biodiversity need assessing. From 2010 to 2013, observational data were collected in Lao PDR from markets selling wildlife, including information on volume, form, species and price of wildlife; market biosafety and visitor origin. The potential for traded wildlife to host zoonotic diseases that pose a serious threat to human health was then evaluated at seven markets identified as having high volumes of trade. At the seven markets, during 21 observational surveys, 1,937 alive or fresh dead mammals (approximately 1,009 kg) were observed for sale, including mammals from 12 taxonomic families previously documented to be capable of hosting 36 zoonotic pathogens. In these seven markets, the combination of high wildlife volumes, high risk taxa for zoonoses and poor biosafety increases the potential for pathogen presence and transmission. To examine the potential conservation impact of trade in markets, we assessed the status of 33,752 animals observed during 375 visits to 93 markets, under the Lao PDR Wildlife and Aquatic Law. We observed 6,452 animals listed by Lao PDR as near extinct or threatened with extinction. The combined risks of wildlife trade in Lao PDR to human health and biodiversity highlight the need for a multi-sector approach to effectively protect public health, economic interests and biodiversity.

## Introduction

It is estimated that 72% of emerging zoonotic disease events originate from wildlife [1]. Many of these diseases pose serious risks to human health, as demonstrated by the 2014 Ebola virus disease (EVD) outbreak in West Africa. Trade that brings wildlife into close proximity with humans and domestic animals provides an interface for pathogen transmission. This interface can contribute to disease emergence, as illustrated by the role of wildlife trade in the spread of a suite of diseases including Severe Acute Respiratory Syndrome (SARS), monkey pox and highly pathogenic avian influenza H5N1 [2–5]. The 2003 SARS outbreak may have been facilitated by wildlife markets in China, as animal traders were found to have higher levels of exposure to SARS-coronavirus than control populations [6]. The disease spread to 29 countries in five continents, leading to 8,098 human cases and 774 deaths. The event was estimated to cost China’s economy $16.8 billion in lost tourism revenue [7,8].

The Lao People’s Democratic Republic (Lao PDR) is home to a rich diversity of wildlife, including mammal, reptile, bird and amphibian species of national or global importance along with high rates of endemism [9]. However, populations are increasingly threatened by high levels of hunting for domestic and international wildlife trade and by habitat loss [9]. Wildlife throughout Lao PDR is declining with many populations now at alarmingly low levels [9]. Wild sources of protein have been an important dietary component for some communities in Lao PDR for generations, but the scale of this practice was relatively small, and limited to subsistence consumption. However, beginning in the early 1980s wildlife began to be traded in wet markets (where live animals or fresh meat are sold, as in this study) and gained momentum after 1986 with the economic opening of the country following inception of the New Economic Mechanism [10], suggesting there is an appetite for wildlife in Lao PDR that is unrelated to subsistence consumption. Wildlife is primarily sold for food, but also for traditional medicine, pets and ornaments [10]. The high volume of wildlife and diversity of species traded in Lao PDR markets has been documented [11–14], but no studies have evaluated the human health risk posed by such trade.

We assessed the potential for zoonotic pathogens to be transmitted from wildlife to humans at markets in Lao PDR by looking at the following: Factor 1 –potential for wildlife and human contact (based on observed volume of wildlife in markets), Factor 2 –potential for traded wildlife to carry a zoonotic pathogen (based on observed wildlife taxa traded and their previous documentation to be a host of a zoonotic pathogen) and Factor 3 –opportunities for pathogen transmission from infected wildlife to humans (based on observed biosafety practices or lack thereof in markets). We then assessed Factor 4 –potential for human spread of a disease from markets to wider populations (based on market location and origin of market visitors). To provide a combined One Health assessment of the impact of wildlife trade on public health and biodiversity, we included an assessment of conservation and socio-economic implications of wildlife trade in Lao PDR, by examining protection status and price of traded wildlife.

## Methods

**Fig 1:**
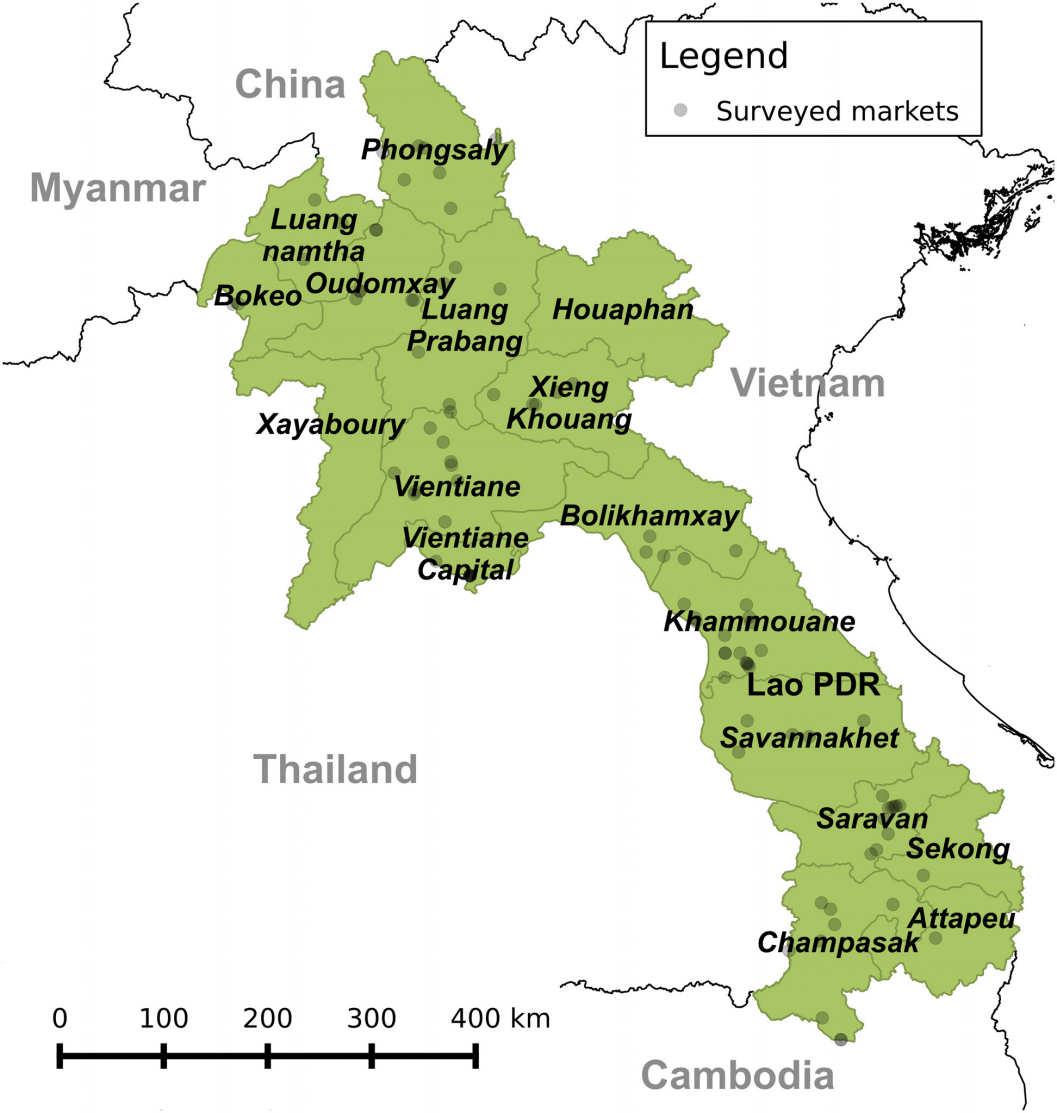
Locations in Lao PDR where basic observational wildlife trade surveys were undertaken between 2010 and 2013. (Created with QGIS v2.6.1-Brighton software and map data from OpenStreetMap contributors).

The research was completed under the approval of the Department of Livestock and Fisheries, Government of Lao PDR. Two observational data collection activities were implemented: a basic market survey and a detailed observational market survey. The basic market surveys were conducted between 2010 and 2013, in 15 of the 17 provinces of Lao PDR. Each basic survey documented one visit to a market and aimed to evaluate the volume of wildlife trade and the types of wildlife being traded. A basic survey targeted all vendors and typically required a half-day to complete, depending on the number of vendors. A total of 375 basic market surveys were conducted at 93 markets where provincial government officials reported that wildlife was sold, as well as sites opportunistically identified (e.g. trade on small roadside markets observed while travelling). Fig 1 shows the locations of market surveys and S1 Fig shows the timeline and visit frequency of surveys. These data enabled the identification of markets where wildlife trade repeatedly occurred and provided the basis for targeting of the detailed market observational surveys.

Detailed market surveys were conducted at 44 markets from February to April 2012. The detailed market surveys, conducted over a minimum of two days, used newly developed structured paper-based data collection tools to guide observations (S1 Methods). Observational data was collected on general market characteristics, products, prices, hygiene of market butchers and vendors and origins of market visitors. Detailed surveys from seven markets with the highest volume of wildlife observed are presented within this manuscript. S1 Methods provides a summary of the different observational surveys conducted, sample sizes and information on the data collection tools used in the market surveys.

For all surveys, visits to markets were not announced and two Lao PDR nationals, who had received in depth training in wildlife species identification, administered the strictly observational surveys while posing as shoppers. The observer periodically left the area of the stalls (e.g. returning to the privacy of the project vehicle) to record findings before returning to the stalls. In most markets, wildlife was openly displayed, but in those where vendors hid wildlife, the observer would remotely watch the vendor until a buyer approached and the vendor brought out the wildlife to sell.

To assess the potential for zoonotic pathogens to be transmitted from wildlife to humans and for pathogen spread (Factors 1, 2, 3 and 4), we focused on analyzing data from seven high volume markets, termed Markets A to G, where the highest wildlife volumes were observed (>100 animals/day on four or more basic survey visits). To assess Factors 1 and 2, we analyzed data from repeated application of the basic survey (three randomly selected surveys; two dry season and one wet season, each a minimum of three months apart) (S2 Fig). To assess Factors 3 and 4, we analyzed data from seven detailed market surveys, one from each of the seven high volume markets.

To assess Factor 1, potential for wildlife and human contact (based on volume of wildlife in markets), the number of whole carcasses of wildlife species or estimated weight of body parts being sold and the condition of the item (live, fresh dead, dried, fermented, frozen, pickled or smoked) was recorded during the basic market surveys. If animals were sold in parts, the market observer calculated the minimum number of individual animals from which the parts could have originated. Frogs, fish and insects were not recorded as part of the study. It was assumed that each animal observed represented a minimum of one contact event between the trader and the animal. Additional contact between wildlife and buyers was observed but was not quantified. A carcass was recorded as fresh if no early signs of decomposition were observed (color changes of skin or fixed lividity, early putrid odor or early skin slippage [15–17]) and had no evidence of freezing.

Only fresh dead or live animals were considered when assessing zoonotic risk. Fresh dead wildlife was included based on previous evidence that; a) fresh carcasses can carry infective viral pathogens, as demonstrated by human outbreaks of EVD, initiated by the handling of primate and bat carcasses [18,19] and b) viral survival without the requirement of a living host can occur in laboratories, for between 24 hours and six days, at temperatures similar to those found in Lao PDR [20–22]. Smoked, dried, fermented and frozen carcasses were excluded due to their unknown potential to be able to transmit pathogens.

To assess Factor 2, the potential for traded wildlife to carry a zoonotic pathogen (based on wildlife taxa traded and their previous documentation to be a host of a zoonotic pathogen), the wildlife species being sold were recorded during the basic market surveys. If the animal being sold could not be identified to species level, the taxonomic family, order or class was recorded and a photo was taken to aid identification later. The mammalian taxonomic families being traded were then analysed for their ability to host zoonotic pathogens that can cause significant human illness or death (hereafter termed ‘significant zoonoses’). To identify the zoonotic pathogens known to occur in each wildlife mammalian taxonomic family, the zoonoses listed in the technical appendices of Levison et al. [23] and Pavlin et al. [24] were summarized to the level of taxonomic family and matched with the taxonomic families observed traded in Lao PDR. Due to insufficient historic surveillance and in order to avoid *a priori* assumptions, the list of significant zoonotic pathogens was not limited to those previously identified in South East Asia (SE Asia). We focused on mammalian hosts because of the frequent previous zoonoses reported from this taxonomic class [23]. Due to lack of host specificity of many infectious organisms, if a pathogen had previously been found in a different genus from the one we observed, but within the same taxonomic family, the genera was recorded as a potential host [24].

To assess Factor 3, opportunities for pathogen transmission from infected wildlife to humans (based on biosafety in markets), data were collected during the detailed market surveys on general market characteristics, including layout and presence of running water (on day one), product(s) displayed by vendor (starting on day one) and hygiene and hand washing practices of market butchers and vendors (half-hour observations of one vendor and one butcher, if present, daily starting on day two). These data were used to identify the presence of hand washing by wildlife vendors, wildlife butchering in markets, cleaning of wildlife butchering tables, running water in markets, meat product contamination on the ground, contact between wildlife and other fresh food products and zoning of domestic meat away from wildlife. Practices of good hygiene during butchery were defined as cleaning of instruments and butcher’s table after each animal, to avoid potential mixing of pathogens between individuals.

To assess Factor 4, potential for human spread of a disease from markets to wider populations (based on market location and origin of market visitors), data were collected during the detailed markets survey on whether the market was located in a city, town or village and whether the market was located along a major road. License plate data of vehicles parked at the market were recorded, to assess the origin of market visitors. Local visitors were defined as having license plates from within province, regional as license plates from outside the province and foreign as license plates from outside Lao PDR.

Finally, we investigated the conservation and socio-economic implications of wildlife trade. We examined the conservation implications of wildlife trade by assessing the protection status of wildlife observed during the basic market surveys, according to the Lao PDR Wildlife and Aquatic Law and the International Union for Conservation of Nature (IUCN) Red List. Whether wildlife was purchased as a luxury versus subsistence food was examined through comparing the price of wildlife to a domestic commodity (pork). Data on the price of wildlife were collected during the detailed market surveys by listening to prices given to buyers. Price was recorded in Lao PDR Kip currency or converted from Thai Baht to Kip for analysis.

## Results

### Factor 1 –Potential for wildlife and human contact

**Fig 2:**
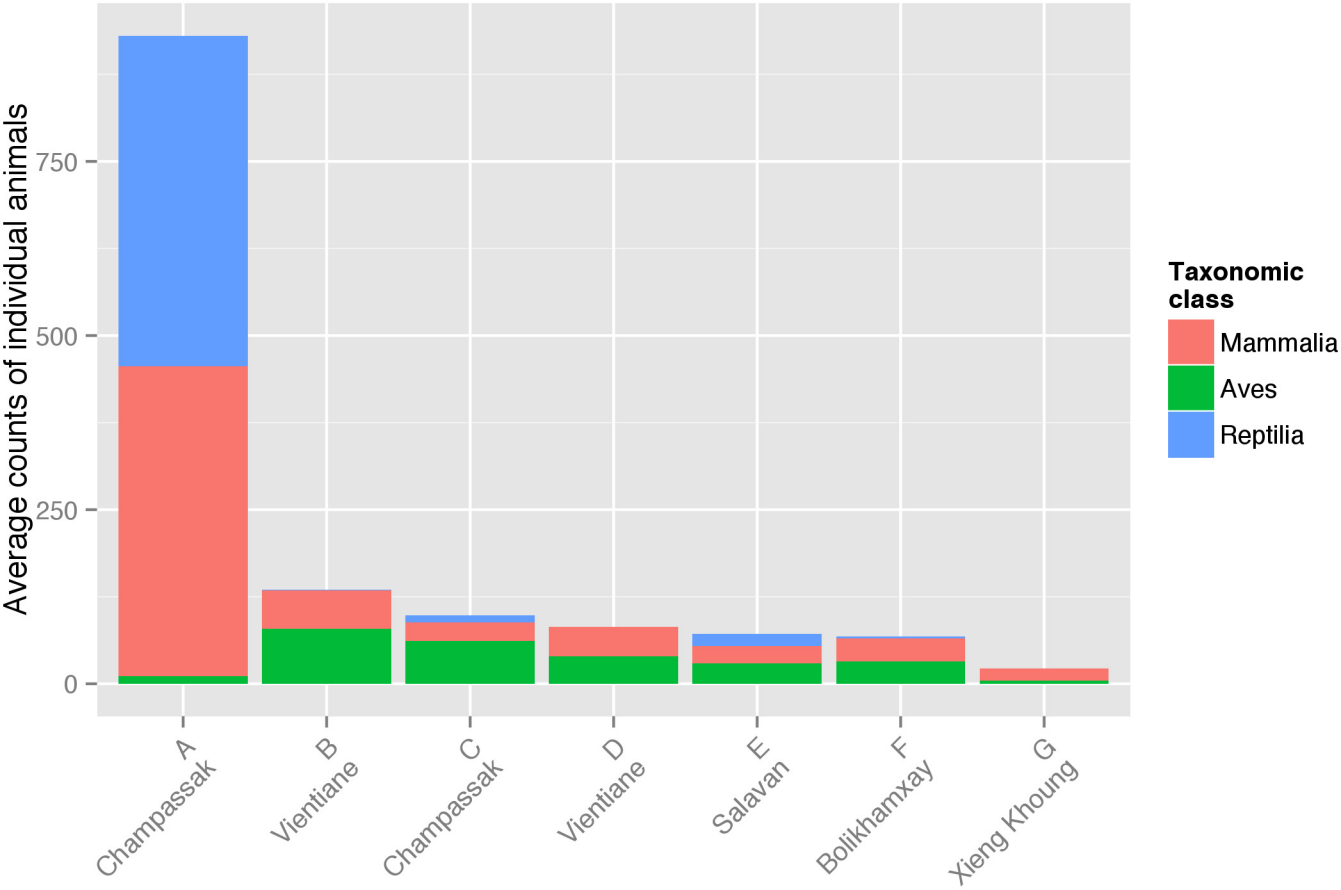
Average number of alive or fresh dead animals per day for markets A-G. Mammals (Mammalia), wild birds (Aves) and reptiles (Reptilia) are shown. The seven high volume markets are listed and Lao PDR province for each corresponding market are provided.

At the seven high volume markets, during 21 surveys, 6,609 individual wild animals or an estimated 2,066 kg of biomass were observed for sale. Of this number, there were 2,021 individual mammals (30.6%), 3,074 birds (46.5%) and 1,514 reptiles (22.9%). Of the mammals, 1937 individuals (approximately 1,009 kg) were alive (53.1%) or fresh dead (42.7%). Of the fresh dead mammals observed, 97.7% were sold whole, 2.3% cut into pieces. The average daily counts of alive or fresh dead animals in these markets ranged from 22 to 931 wild animals per day, with average alive and fresh dead mammal counts of 18 to 445 animals/day (Fig 2). The volume of wildlife for sale varied greatly between surveys at these seven markets. A Student’s t-Test found no significant difference between wet and dry seasons for the mean of counts (p = 0.1958) or masses (p = 0.2746) of wild animals.

### Factor 2 –Potential for traded wildlife to carry a zoonotic pathogen

A variety of mammalian, avian and reptilian taxa were observed in markets (Table 1, Fig 2). On an average day, at a typical market, a visitor or worker would likely encounter rodents, ungulates, carnivores, wild birds, bats (if near limestone karsts) and lizards (if in the south of Lao PDR). In the seven high volume markets, a range of 1–23 genera/day (mean 9 genera) were observed. The most to least commonly observed mammalian orders (by individual animals) were Rodentia, Chiroptera, Artiodactyla, Carnivora, Lagomorpha, Scandentia, Primates and Dermoptera.

**Table 1:** Surveillance summary.

Order	Total number of individuals of order observed	Total biomass (kg) of order observed	Number of sites where order was observed	Number of visits when order was observed	Family	% of individuals by family	Common name of most frequently observed species
Passeriformes	2,714	109	7	14	Hirundinidae	85	martin species
					Pycnonotidae^1^	14	bulbul species
Rodentia	1,698	625	7	20	Sciuridae^2^	83	tree squirrels and flying squirrel species
					Muridae^3^	12	rat species
					Spalacidae^4^	4	bamboo rat species
Squamata	1,508	800	5	11	Agamidae^5^	93	crested lizard
					Varanidae^6^	5	monitor lizard
Chiroptera	187	8	5	8	Pteropodidae^7^	55	fruit bat species
					Unknown	32	insectivorous bat species
					Rhinolophidae^8^	13	insectivorous bat species
Galliformes	65	54	7	16	Phasianidae^9^	100	junglefowl, partridge, francolin and pheasant species
Artiodactyla	49	216	5	10	Tragulidae^10^	67	mouse deer
					Cervidae^11^	24	muntjac and sambar species
					Suidae^12^	8	wild boar
Carnivora	45	141	7	14	Viverridae^13^	87	civet species
					Herpestidae^14^	9	Mongoose
					Felidae^15^	4	leopard cat
Lagomorpha	24	55	2	4	Leporidae^16^	100	burmese hare
Psittaciformes	19	1	2	2	Psittaculidae^17^	100	parakeet species
Anseriformes	16	10	2	2	Anatidae^18^	100	duck species
Columbiformes	15	3	5	7	Columbidae^19^	100	dove and pigeon species
Scandentia	15	3	5	6	Tupaiidae^20^	100	northern tree shrew
Primates	2	2	2	2	Lorisidae^21^	100	Asian slow loris
Dermoptera	1	1	1	1	Cynocephalidae^22^	100	Sunda flying lemur

Data based on 21 visits to seven markets showing taxonomic orders and families observed. Taxonomic families listed make up 98% or more of each order. Genera observed are referenced in superscript and are listed under the table. Non-mammalian orders that were seen in very small volumes (less than 10 individual animals) are excluded (Testudines, Strigiformes, Cuculiformes, Pelecaniformes, Charadriiformes, Coraciiformes, Accipitriformes and Piciformes). Genera observed were:

^1^Pycnonotus;

^2^Callosciurus, Dremomys, Hylopetes, Menetes, Petaurista, Ratufa;

^3^Leopoldamys, Niviventer;

^4^Rhizomys;

^5^Lophocalotes, Physignathus;

^6^Varanus;

^7^Megaerops;

^8^Rhinolophus;

^9^Arborophila, Gallus, Lophura, Polyplectron;

^10^Tragulus;

^11^Muntiacus;

^12^Sus;

^13^Paradoxurus, Viverra, Viverricula;

^14^Herpestes;

^15^Prionailurus;

^16^Lepus;

^17^Psittacula;

^18^Dendrocygna;

^19^Chalcophaps, Spilopelia, Treron;

^20^Tupaia;

^21^Nycticebus;

^22^Galeopterus.

**Fig 3:**
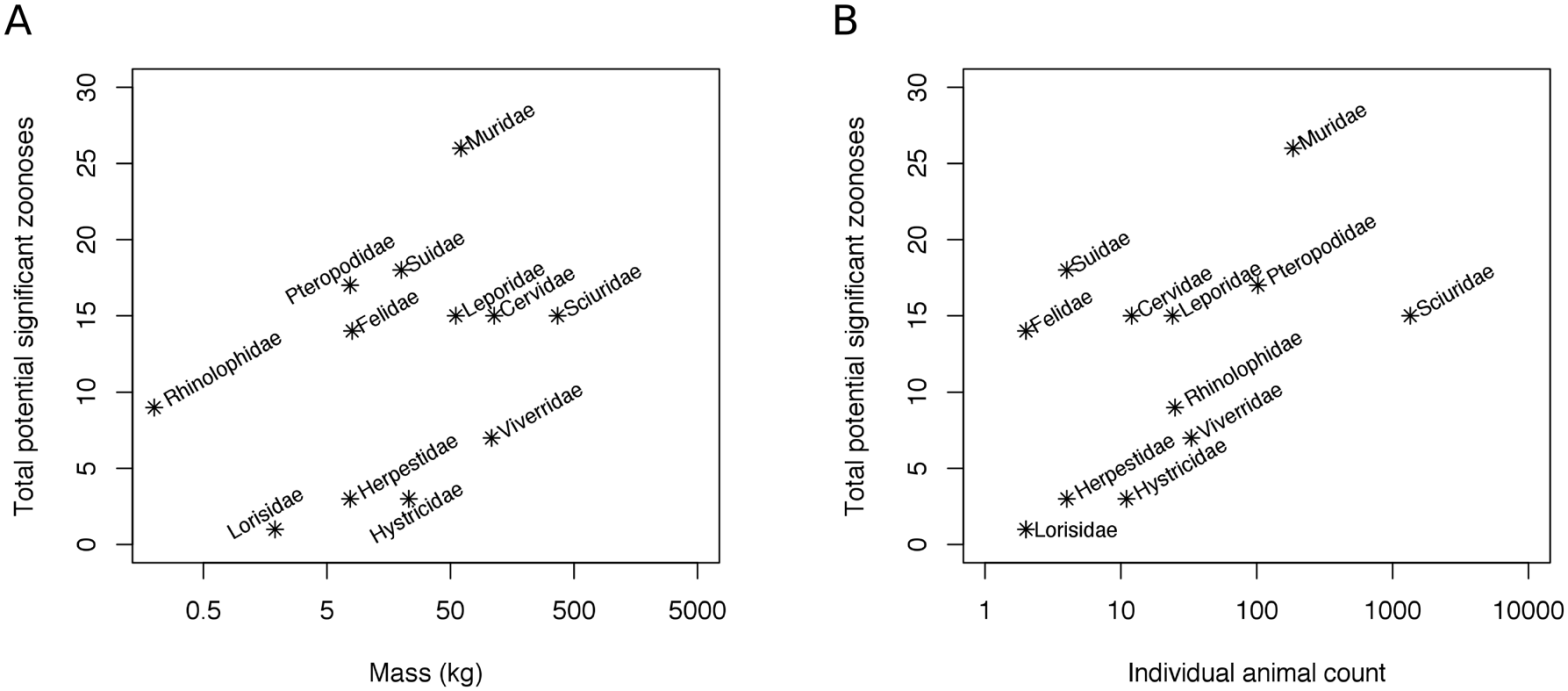
Number of potential significant zoonoses per mammalian family based on total live and fresh dead mass (A) and individual animal count (B) of mammals. Individual animal count and biomass (kg) observed for seven markets over 21 visits on a log scale are shown. Only families capable of hosting one or more zoonosis are represented. Precise biomass and counts are provided in the S1 Table.

For mammals that were live or freshly dead, 21 genera from 12 wildlife families were observed that have the potential to host 36 significant zoonotic pathogens, including those associated with diseases such as rabies, SARS, leptospirosis and Mycobacterium tuberculosis complex (for list of significant zoonoses, see Table 2; for breakdown by taxonomic family see S1 Table). The 12 wildlife families were Muridae (rat species; potential to host 26 significant zoonoses), Suidae (wild pig; 18), Pteropodidae (fruit bats; 17), Sciuridae (tree and flying squirrels; 15), Cervidae (muntjac, sambar; 15), Leporidae (hare, 15), Felidae (leopard cat; 14), Rhinolophidae (insectivorous bats; 9), Viveridae (civets; 7), Herpestidae (mongoose; 3), Hystricidae (porcupine; 3) and Lorisidae (loris; 1). Fig 3 shows there were five mammalian families seen in large volumes (greater than 100 individuals or kg per family) that are capable of hosting a high number (7 to 26) of significant zoonoses: Sciuridae, Pteropodidae, Muridae, Cervidae and Viverridae.

**Table 2:** List of significant zoonoses capable of infecting mammals globally, for which a potential wildlife host was identified in Lao PDR.

Pathogen type	Zoonotic disease
Viral diseases (Non-vector borne)	Crimean-Congo hemorrhagic fever virus, Ebola viruses, Hantaviruses associated with HCPS, Hantaviruses associated with HFRS, Hendra virus, Hepatitis E virus, highly pathogenic avian influenza virus (H5N1), Lassa fever virus, Lymphocytic choriomeningitis virus, Marburg virus, Monkeypox virus, Nipah virus, Rabies viruses, Rift Valley fever virus, Rotavirus B, SARS virus (or SARS-like CoV), South American hemorrhagic fever arenaviruses
Viral diseases (Vector-borne)	California encephalitis, Chikungunya virus, Dengue virus, Eastern equine encephalitis virus, Japanese encephalitis virus, St. Louis encephalitis virus, Tick-borne encephalitis virus complex, Venezuelan equine encephalitis virus, West Nile virus, Western equine encephalitis virus, Yellow fever virus
Parasitic diseases	*Echinicoccus spp*
Bacterial diseases	*Bacillus anthracis*, *Brucella spp*., *Coxiella burnetii*, *Francisella tularensis*, *Leptospira spp*., *Mycobacterium tuberculosis complex*, *Yersinia pestis*

Only known viral, parasitic, fungal and bacterial zoonoses that cause severe disease or death in humans are included. Diseases considered as significant zoonoses are based on Pavlin et al. [24] and Levison et al. [23]. See S1 Table for more details.

### Factor 3 –Opportunities for pathogen transmission from infected wildlife to humans

In the seven high volume markets, potential for transmission of pathogens from infected wildlife to humans, directly or indirectly was observed (Table 3). Routes for direct transmission included the handling and butchering of wildlife by vendors and, at one market (Market A), through butchering and consumption of wildlife by the public on the premises, including the traditional consumption practice of eating fast grilled un-gutted wildlife. In half-hour observations of 11 wildlife vendors in these seven markets, hand washing was only observed to be performed by one individual. As wildlife was generally sold whole, the presence of wildlife butchering was only observed in four of the seven markets. Potential indirect transmission routes observed were contaminated fomites and cross contamination of food. The risk of contaminated fomites was increased, as only four of the seven markets had running water, and of six markets where domestic animal meat was sold, all were found to have areas with either animal blood or entrails on the ground. During five half-hour observations of five butchers, none were observed to clean instruments and only one was observed to clean the work table. The level of contact of wildlife with other fresh food products (and therefore potential for cross contamination) was high in most markets. Wildlife was observed for sale on stalls also displaying vegetables or other fresh produce, in five of the seven markets. Although zoning was seen in five of the six markets selling domestic animal meat (defined as domestic pork and beef being sold in an area of the market separate from stalls selling wildlife), this separation did not necessarily apply to poultry and fish, which were observed interspersed throughout areas of the market where wildlife was located.

**Table 3:** Summary for the seven high volume markets of key factors affecting the potential for a zoonotic disease to be transmitted from wildlife to humans and spread of a disease outbreak from markets to wider human populations.

Market	Alive or fresh dead wildlife count/day		Alive or fresh dead mammals from families with potential to host 1 or more significant zoonoses count/day		Zoning of domestic red meat	Running water	Dirty floor or substrate (either blood or entrails on floor)	Wildlife vendor–hand washing^a^	Wildlife butchering present^a^	Location of market in town (T), on major road (R) or neither (N)	Local (L), other regions in Lao PDR (R), foreign (F) license plates
	Mean	SD	Mean	SD							
A	931	1190	436	469	NA	N	NA	N	Y	N	L
B	135	93	51	24	N	N	Y	Y,N,N	N,N,N	N	L,R
C	98	118	26	22	Y	N	Y	N	Y	R	L,R
D	82	120	28	30	Y	Y	Y	N	N	T,R	L,R
E	71	43	24	10	Y	Y	Y	N	N	T	L
F	68	52	36	23	Y	Y	Y	N	Y	T,R	L,R
G	22	15	8	9	Y	Y	Y	N,N,N	Y,N,Y	T	L,R,F

^a^Multiple responses indicate that more than one individual was observed at that market over the course of the detailed survey visit.

### Factor 4 –Potential for human spread of a disease from markets to wider populations

Five of the seven high volume markets were located either in towns or on major roads. From vehicle license plate data, two markets had only local customers; four had local and regional customers; and one had local, regional and foreign customers (see Table 3). License plate data, however, does not necessarily reflect the origin of all customers. International visitors (western tourists, Vietnamese, Chinese, Thai or Korean) were observed by field teams inside all seven markets on at least one occasion during surveys. Table 3 provides a summary, for the seven high volume markets, of risk factors for a zoonotic disease to be transmitted from wildlife to humans and spread to wider human populations.

### Conservation and socio-economic implications of wildlife trade: protection status and price of traded wildlife

Of the 33,752 animals observed during 375 visits to 93 markets, we were able to report the protection status of 6,452 individuals (S2 Table). Under the Lao PDR Wildlife and Aquatic Law, there were 382 Category I animals, species classified as being currently rare or near extinct, and 6,070 Category II animals for which, if management is neglected, they will become extinct. A total of 286 animals from 30 species were observed that are classified as threatened on the IUCN redlist, including Critically Endangered (1 individual), Endangered (31), Vulnerable (206) or Near Threatened (48) (S3 Table).

**Fig 4:**
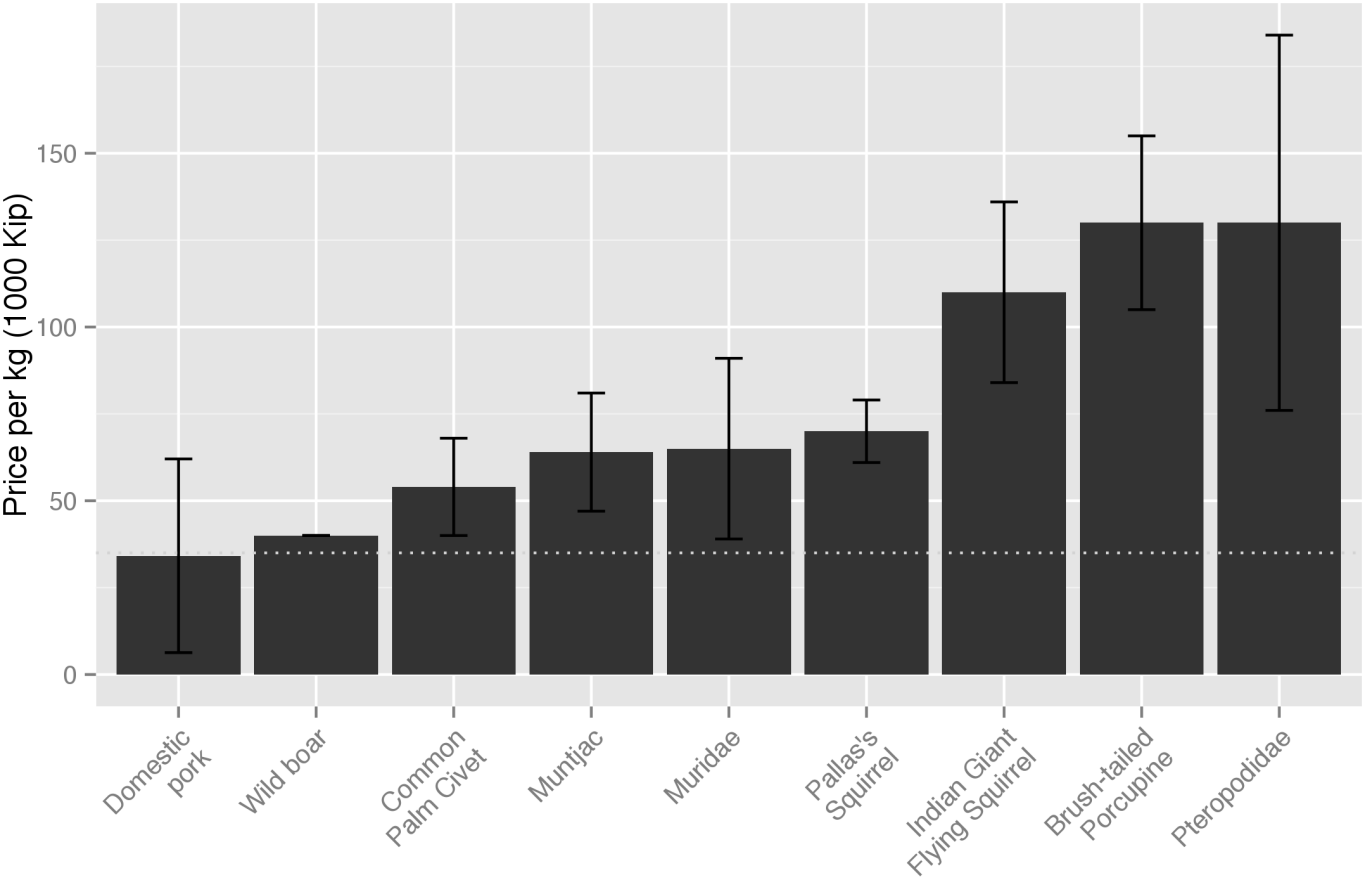
Comparison of average price of fresh dead wildlife (February–April 2012) to the price of domestic pork in Lao PDR. Bars represent standard deviation. Where wildlife was priced per individual, price was converted to Kip/kg using average body weight for wildlife species. The average price was based on observations of sales of: nine common palm civet, 33 Pallas’s squirrel, 14 Indian giant flying squirrel, 17 Pteropodidae, 40 Muridae and six domestic pork samples. For sales observations of the brush-tailed porcupine (n = 10), muntjac (n = 10) and wild boar (n = 3), wildlife was priced per kilogram. The price of rice (used as an indicator for the expected level of price variation across the country) did not vary significantly between vendors in markets or between different markets; the average across all markets was 4,982 Kip/kg.

On average, fresh dead wildlife was consistently more expensive than an equivalent amount of fresh dead domestic pork. The average price recorded for pork in early 2012 was 34,000 Kip/kg (SD 28,000), while brush-tailed porcupine sold at an average price of 130,000 Kip/kg (SD 25,000), muntjac at 64,000 Kip/kg (SD 17,000) and wild boar at 40,000 Kip/kg (SD 0). At the time of data collection, 8,000 Kip equaled approximately 1 US dollar ($US). For wildlife priced per individual (common palm civet, Pallas’s squirrel, Indian giant flying squirrel, Pteropodidae and Muridae) and converted to kilograms, using average mass, wildlife was again higher in price per kilogram than domestic pork (Fig 4).

## Discussion

### Factor 1 –Potential for wildlife and human contact

This study demonstrates that the magnitude of domestic wildlife trade in Lao PDR markets is high and that there is significant potential for contact between wildlife and humans. This contact includes high-risk interactions with alive and fresh dead mammals, of which 1,937 specimens were observed in the seven high volume markets. As we have assessed wildlife volume in markets using observational methods, our counts are likely to underestimate the true volume of wildlife present, as sellers may not display all items on offer. However, due to the lack of enforcement to curb illegal wildlife, in the seven high volume markets at the time of the study, wildlife was mostly displayed openly and thus underestimates are unlikely to be large. In another market where regular enforcement activities had occurred, the vendors were wary and tended to hide their goods. However this market was an anomaly.

Repeated observations in the seven high volume markets support the conclusion that average daily counts of wildlife in markets in Lao PDR are equivalent in scale to markets considered as significant hubs of bush meat trade globally. The average daily counts of alive or fresh dead animals in the seven high volume markets ranged from 22 to 931 wild animals per day. This rate is similar to those found in the two most prolific wildlife market towns in Equatorial Guinea (18,012 carcasses observed over 424 markets days, an average of 42 wild animals/day) [25] and levels reported at a trade hub in Myanmar exporting wildlife from Myanmar to China (179 wild animals/day) [26]. Our figures were only lower than those reported in four major wildlife markets in Guangzhou and Shenzhen, the largest wildlife consuming provinces in China [27,28].

This study did not examine international wildlife trade bypassing local markets, thus large-scale trade of species such as bears, tigers, turtles, pangolins and birds is missing from the analysis. Such trade represents further potential for contact between humans and wildlife in Lao PDR.

### Factor 2 –Potential for traded wildlife to carry a zoonotic pathogen

The mixture of genera from a range of taxonomic classes (mammals, birds and reptiles), suggests that there is potential for a high diversity of pathogens to be present in certain markets. The trade of mammals (21 genera from 12 families) that are capable of hosting 36 significant zoonoses (based on global human pathogen data), including diseases such as rabies, SARS, leptospirosis and Mycobacterium tuberculosis complex, has the potential to pose significant public health risk. With the addition of the zoonoses associated with reptiles and wild birds [29–32], the potential for wildlife in markets to harbor zoonotic pathogens increases. Our assessment correlates risk with the presence of a genus known to carry a globally significant zoonosis. While we are likely overestimating the true potential of any given species within a genus to host a specific zoonosis, we are also likely underestimating any unknown zoonosis that may be specific to the region or a species. The high volumes of Sciuridae (tree squirrels and flying squirrels), Muridae (rats), Pteropodidae (fruit bats), Cervidae (muntjac and sambar) and Viverridae (civets) are of particular concern, as these families host large numbers of significant zoonoses (between 7 and 26) and are reservoir hosts for several zoonoses that have already been identified in Asia; rats are the reservoir hosts of almost all arenaviruses and hantaviruses [33,34]; bats have been implicated as reservoir hosts for Nipah virus in Bangladesh and Malaysia [35,36], for SARS in China [37–39] and recently as a possible reservoir for Ebola virus or Ebola-like viruses in mainland Asia [40].

The potential risk for transmission of zoonotic pathogens in Lao PDR reflects similar risks documented in Africa from the bush meat trade and in studies assessing the health risks associated with the import of wildlife into the US. The high prevalence of fruit bats recorded in trade in Ghana was similar to our findings in markets in Lao PDR. In Ghana, researchers found a minimum of 128,000 fruit bats were traded in a year, with the majority passing through marketplaces [41]. The study highlighted the risk such trade could pose for zoonoses, such as henipaviruses, lyssaviruses and Ebola virus. In Guinea, the 2014 Ebola outbreak may have stemmed from exposure of a boy to bats, through hunting or playing near a roost [42], and has further highlighted the risk of bat hunting and trade in Africa. The rodent trade, commonly observed in Lao PDR, also occurs in Ghanaian markets [43]. While no assessment has been made of the zoonotic risk posed by the rodent trade to the people of Ghana, an international risk was demonstrated in 2003 when a monkeypox outbreak occurred in humans in the US following the import of infected rodents from Ghana [2]. The ‘myriad opportunities’ that wildlife trade provides for importing a zoonotic pathogen into the US [24] was further demonstrated by Smith et al. [44] who detected retroviruses (simian foamy virus) and herpesviruses (cytomegalovirus and lymphocryptovirus) in non-human primate samples being imported into the US. Researchers have also found a substantial proportion of primates being sold as bush meat or kept as pets in Cameroon were infected with Simian immunodeficiency virus, posing a potential source of infection to humans who hunt or handle bush meat [45]. In Lao PDR we only observed primates to be traded in small volumes through markets, as they are taxa more likely to be traded outside the market setting, for pets or macaque breeding farms.

### Factor 3 –Opportunities for pathogen transmission from infected wildlife to humans

In all seven high volume markets, poor biosafety enhanced the opportunities for transmission of pathogens from wildlife to humans. Lack of hand washing and cleaning of tables and generally poor market cleanliness, combined with the practice of selling wildlife alongside other fresh produce, presents risks for food contamination and infection of humans with pathogens, either directly or indirectly. This risk is increased in markets where wildlife butchering occurs. In Market A, where fast grilled un-gutted wildlife was consumed on site by the public, the risk of insufficient cooking and associated pathogen transmission could be high.

The detailed survey visits were not repeated at the large markets and so only offer a glimpse at practices which may be impacted by seasonal events, providing an entry point for more focused research.

### Factor 4 –Potential for human spread of a disease from markets to wider populations

The location of the majority of the high volume markets in towns or on major roads combined with the observation of foreigners in markets and frequent observation of license plates from other regions of Lao PDR documented a risk for disease spread both nationally and internationally.

### Conservation and socio-economic implications of wildlife trade: protection status and price of traded wildlife

The observed 6,452 animals listed as near extinct or threatened with extinction, under the Wildlife and Aquatic Law, are likely to represent only a fraction of the endangered wildlife being traded during the study period, as we only conducted surveys during 125 (1.55%) of the 8,055 potential trade days for the seven high volume markets. Trade has already led to significant declines of wildlife populations and endemic biodiversity in Lao PDR, with populations of large-sized mammals decreasing significantly from the early 1980s and small mammals declining since the 2000s [10]. The high volumes of trade of Lao PDR Category I and II species observed to be occurring are likely to perpetuate these declines. Of particular concern, the trade of Artiodactyla (muntjac, sambar, mouse deer), that are important prey species for carnivores, threaten the survival of iconic species like the tiger.

Although subsistence hunting still exists in many parts of Lao PDR, there are increasing trends of hunting villages selling wildlife for cash income [14]. Our findings that the price of wildlife meat is often higher than domestic animal meat support the suggestions of others that wildlife is increasingly sold as luxury food, rather than for subsistence, and that wildlife is increasingly bought by urban consumers [10,12,13,46,47]. The average GNI per capita in Lao PDR is US$1,460 [48], equating to a daily income of US$4. Using the market prices recorded in this study, one kilogram of bat equates to over three days income, verses one day of income for a kilogram of domestic pork. Cash income generated from wildlife sales can be important for rural households in Lao PDR, contributing in some areas up to 6% of income generated from sale of non-timer forest products [49,50]. However, concurrently, overhunting of wildlife threatens food security, as wildlife consumed by local hunters acts as an important subsistence protein source [51]. This highlights the need for sustainable wildlife harvest, as set out in the Lao PDR Wildlife and Aquatic Law.

## Conclusion

This study was conducted in collaboration with stakeholders from the veterinary, public health and conservation sectors. The data presented on the volume and species of wildlife and biosafety found in markets in Lao PDR demonstrate that there are significant opportunities in certain markets for wildlife, and any zoonotic pathogens they carry, to come into contact with humans. The large number of individual wild animals from high risk taxa for carrying zoonoses, poor biosafety and potential for disease spread through the movement of regional or international market visitors are all risk factors for the occurrence of a disease emergence event similar to the public health catastrophes of SARS and EVD. The volume, scale and diversity of wildlife moving through markets in Lao PDR also represent a serious threat to wildlife conservation.

Markets have and will likely continue to play a role in disease emergence if wildlife trade is not controlled and market management practices are not improved. The double risk of wildlife trade to public health and the conservation of Lao PDR’s rich biodiversity represent a unique opportunity for multiple agencies and stakeholder groups (including law enforcement officers, commerce and trade authorities, public health inspectors and veterinarians) to join forces and address a common threat.

## Disclaimer

The contents are the responsibility of the authors and do not necessarily reflect the views of United States Agency for International Development, National Institutes of Health, or the United States Government.

## Supporting Information

S1 Database Raw data from basic and detailed market surveys. (XLSX)

S1 Fig Overview of basic survey visits to the seven high volume markets (A:G; n = 125) and all other markets (M01:M86; n = 250). (DOCX)

S2 Fig Sampling timeline of basic market surveys at the seven high volume markets, used for zoonotic risk analysis. (DOCX)

S1 Methods Summary of observational surveys conducted and analysis conducted on each dataset. (DOCX)

S1 Table Significant zoonoses by taxonomic family. Diseases considered as significant zoonoses are based on Pavlin et al. [24], (marked P in table below), and Levison et al. [23] (marked L in the table). Significant zoonoses jointly reported by Pavlin et al. [24] and Levison et al. [23] are marked with a P. Due to the non-host specificity of many infectious organisms, if a pathogen had previously been found in a different genus from the one we observed being traded, but within the same taxonomic family, the genera was recorded as a potential host [24].
(DOCX)

S2 Table Species observed being traded that are listed in the Lao PDR Wildlife and Aquatic Law. Species included are those classified as Category I, species that are rare or near extinct or Category II, species where if management is neglected, they will become extinct.
(DOCX)

S3 Table Species observed being traded that are listed on the IUCN Redlist. Species included as those classified as Critically Endangered (CR), Endangered (EN), Vulnerable (V) or Near Threatened (NT).
(DOCX)
