# Supplementary material for: Wildlife Trade and Human Health in Lao PDR: An Assessment of the Zoonotic Disease Risk in Markets
Source: PLoS One. 2016 Mar 23;11(3):e0150666. doi: 10.1371/journal.pone.0150666 (PMC4805265; doi:10.1371/journal.pone.0150666)

**S1 Figure. Overview of basic survey visits to the seven high volume markets (A:G; n=125) and all other markets (M01:M86; n=250).** Sampling timeline (A) and histograms of visit frequency to all markets (B) and seven high volume markets (C) are shown. Tightly clustered visits may be obscured but details are provided in S4 Database and S1 Methods.


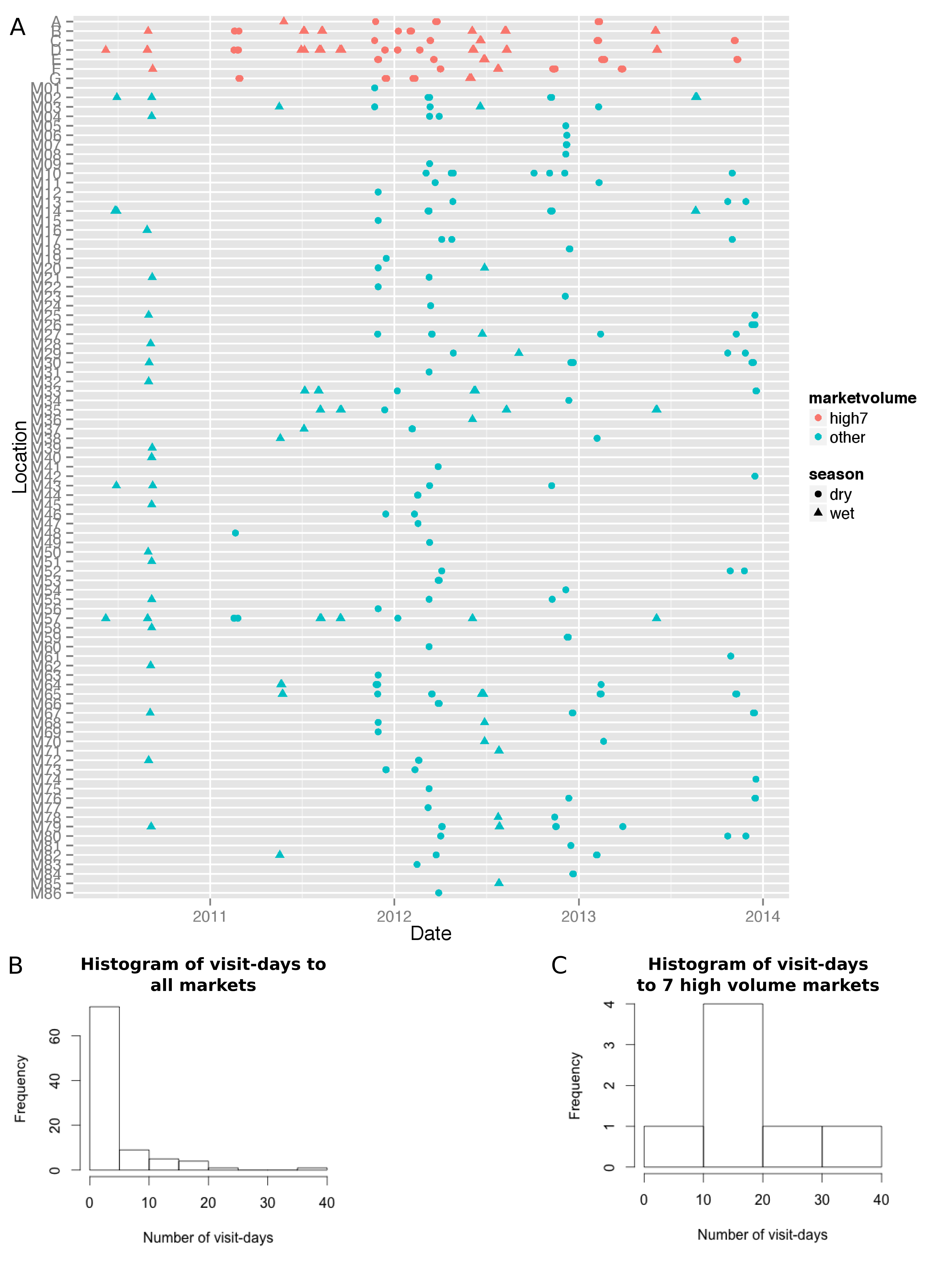

Supplement: S1 Fig — (DOCX) [file pone.0150666.s002.docx]
