# Supplementary material for: Wildlife Trade and Human Health in Lao PDR: An Assessment of the Zoonotic Disease Risk in Markets
Source: PLoS One. 2016 Mar 23;11(3):e0150666. doi: 10.1371/journal.pone.0150666 (PMC4805265; doi:10.1371/journal.pone.0150666)
Supplement: S2 Fig — (DOCX) [file pone.0150666.s003.docx]

**S2 Figure. Sampling timeline of basic market surveys at the seven high volume markets, used for zoonotic risk analysis.**

**
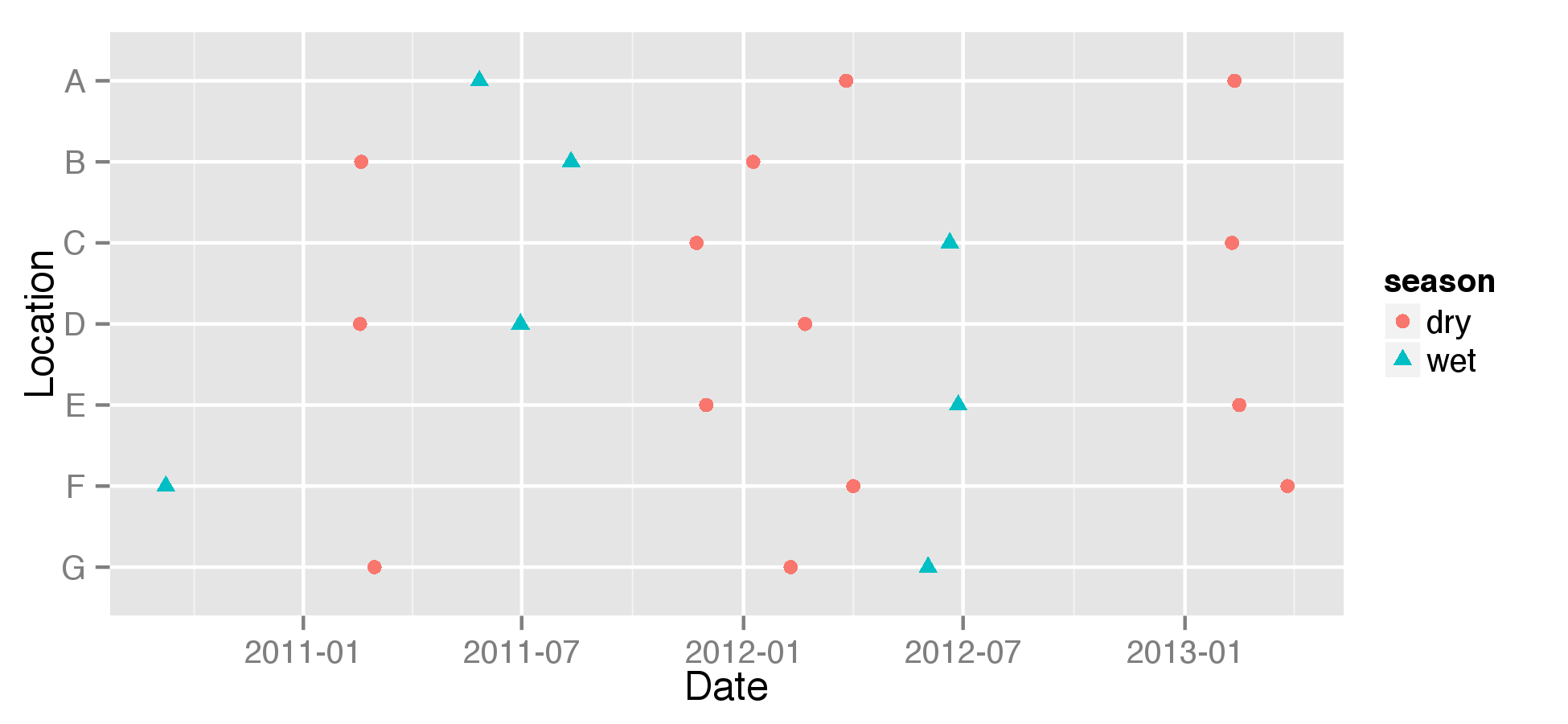
**
