# Supplementary material for: Wildlife Trade and Human Health in Lao PDR: An Assessment of the Zoonotic Disease Risk in Markets
Source: PLoS One. 2016 Mar 23;11(3):e0150666. doi: 10.1371/journal.pone.0150666 (PMC4805265; doi:10.1371/journal.pone.0150666)
Supplement: S1 Methods — (DOCX) [file pone.0150666.s004.docx]

S1 Methods

Summary of observational surveys conducted and analysis conducted on each dataset.

| **Sampling component** | **Basic Market Surveys** | | **Detailed Market Surveys** | |
| --- | --- | --- | --- | --- |
|  | **Entire Dataset** | **Subset of basic market surveys used for zoonotic risk analysis** | **Entire Dataset** | **Subset of detailed market surveys used for Factor 3 and 4 analysis** |
| **Survey Period** | June 2010 – December 2013 | Subset of data from basic market surveys, spanning September 2010 – March 2013 | February 2012 – April 2012 | February 2012 – April 2012 |
| **Location** | 93 markets in 15 of 17 provinces of Lao PDR | 7 high volume markets identified in basic market surveys, in 5 provinces of Lao PDR | 44 markets in 10 provinces of Lao PDR | 7 high volume markets identified in basic market surveys, in 5 provinces of Lao PDR |
| **Sample size** | 375 surveys | 21 basic market surveys. Two dry season and one wet season survey per market, randomly selected. Surveys minimum of 3 months apart | 44 markets were surveyed using a minimum of one and a maximum of four research instruments. | 7 surveys were completed using up to four research instruments per market visit. |
| **Length of survey** | Each survey half day | As per basic market surveys | Data collection lasted between 2 and 4 days per market | Data collection lasted between 2 and 4 days per market |
| **Data collected** | Volume and species of wildlife being traded | As per basic market surveys | General market characteristics, products, prices and volume, hygiene of market butchers/vendors and origins of market visitors | General market characteristics, products, prices, hygiene of market butchers/vendors and origins of market visitors |
| **Analysis of data** | Protection status of wildlife being traded. Identification of 7 high volume markets (>100 animals/day on four or more survey visits) | Factor 1 (potential for wildlife/human contact) and Factor 2 (potential for traded wildlife to carry a zoonotic pathogen) | N/A | Factor 3 (opportunities for pathogen transmission from infected wildlife to humans)  Factor 4 (potential for human spread of a disease from markets to wider populations) and price of wildlife |
| **Additional information** | Figure S1 for detail on timing of surveys | Figure S2 for detail on timing of surveys | N/A | N/A |

For the basic market study, one structured paper-based data collection tool was used as a basis for the observational surveys. For the detailed market study (February – April 2012) five structured paper-based data collection tools were used as a basis for the observational surveys. Each instrument collected different data points used in the final analysis on market hygiene, sanitation, and biosafety practices.

| **Form** | **Observational data collected** | **Preferred time for collecting data** | **Outcome** |
| --- | --- | --- | --- |
| **Basic Market Survey** | | | |
| Wildlife vendor observation | Wildlife species to lowest taxonomic order, number of individuals or kg of that species, form of wildlife (fresh, dry etc) | The form was completed at peak times for each market (based on information from government) | Allow assessment of volume of trade and species being traded |
| **Detailed Market Survey** | | | |
| A: Market general observation  (4 pages) | Size of market (# of vendors and their products); Infrastructure (Water, electricity, place in relation to area); Cleanliness (Toilets, garbage disposal) | Upon arrival at a new market this is the first form completed.  *The form will not be used more than once at any market. | This profile will allow us to categorize the market by size and provide details for future follow-up visits. |
| B: Products sold by vendor  (2 pages) | Details of products sold by each vendor and basic vendor characteristics | Ideally the form was completed early in the morning (or at the time when each vendor is fully stocked for that day) and in the late afternoon, before the market begins closing. | Identify mixing of species and common products sold together (biosecurity and substitutes). |
| C: Pricing table  (3 pages) | Document price and quantity of key species, staples and domestic meat; Shopper preferences and price/quantity information | The form was used when there are many shoppers in the market and prices/quantities can be overheard. | Provides input for future price and quantity monitoring. |
| D: Market actors observation – butcher, vendor, and shopper  (3 pages) | Observe butcher and vendor demographics and sanitation; Observe shopper demographics and preferences | This form was used at the start and end of the day close to market closing, to observe one butcher and one vendor, if present. | Identify characteristics of wildlife butchers, vendors, and shoppers as well as biosafety. |
| E/F: Small market observation – roadside vendor market mapping form  (1 page) | Document small road-side stands/markets that sell key wild animal species. (Name of species available, number of key species sold, number of vendors selling key species) | This form was used whenever the team is on a route they have not documented roadside vendors previously. | Provide a snapshot of informal roadside stands/markets. |
