# Supplementary material for: Wildlife Trade and Human Health in Lao PDR: An Assessment of the Zoonotic Disease Risk in Markets
Source: PLoS One. 2016 Mar 23;11(3):e0150666. doi: 10.1371/journal.pone.0150666 (PMC4805265; doi:10.1371/journal.pone.0150666)
Supplement: S1 Table — Diseases considered as significant zoonoses are based on Pavlin et al. [24], (marked P in table below), and Levison et al. [23] (marked L in the table). Significant zoonoses jointly reported by Pavlin et al. [24] and Levison et al. [23] are marked with a P. Due to the non-host specificity of many infectious organisms, if a pathogen had previously been found in a different genus from the one we observed being traded, but within the same taxonomic family, the genera was recorded as a potential host [24]. (DOCX) [file pone.0150666.s005.docx]

**S1 Table**. **Significant zoonoses by taxonomic family.** Diseases considered as significant zoonoses are based on Pavlin et al. [24], (marked P in table below), and Levison et al. [23] (marked L in the table). Significant zoonoses jointly reported by Pavlin et al. [24] and Levison et al. [23] are marked with a P. Due to the non-host specificity of many infectious organisms, if a pathogen had previously been found in a different genus from the one we observed being traded, but within the same taxonomic family, the genera was recorded as a potential host [24].

| **Taxonomic Family of Potential Host** | **Sciuridae** | **Pteropodidae** | **Muridae** | **Viverridae** | **Rhinolophidae** | **Cervidae** | **Suidae** | **Lorisidae** | **Leporidae** | **Felidae** | **Herpestidae** | **Hystricidae** |
| --- | --- | --- | --- | --- | --- | --- | --- | --- | --- | --- | --- | --- |
| **Biomass (kg) observed (Figure 3A)** | 367.9 | 7.7 | 60.7 | 107.5 | 0.2 | 113.0 | 20.0 | 1.9 | 55.2 | 8.0 | 7.7 | 23.0 |
| **Individual animal count observed (Figure 3B)** | 1349 | 102 | 185 | 33 | 25 | 12 | 4 | 2 | 24 | 2 | 4 | 11 |
| **Viral diseases (non-vector borne)** |  |  |  |  |  |  |  |  |  |  |  |  |
| Crimean-Congo hemorrhagic fever virus | P |  | P | P |  |  | P |  | P | P |  | P |
| Ebola viruses* |  | P | P |  |  |  | P |  |  |  |  |  |
| Hantaviruses associated with HCPS* |  |  | P |  |  |  |  |  |  |  |  |  |
| Hantaviruses associated with HFRS* |  |  | P |  | P |  | P |  |  |  |  |  |
| Hendra virus |  | L |  |  |  |  |  |  |  |  |  |  |
| Hepatitis E virus |  |  | L |  |  | L | L |  |  |  | L |  |
| Highly pathogenic avian influenza virus (H5N1) |  |  | P | P |  |  | P |  |  | P |  |  |
| Lassa fever virus |  |  | P |  |  |  |  |  |  |  |  |  |
| Lymphocytic choriomeningitis virus | P |  | P |  |  |  |  |  |  |  |  |  |
| Marburg virus |  | P |  |  | P |  |  |  |  |  |  |  |
| Monkeypox virus | P |  | P |  |  |  |  |  | P |  |  |  |
| Nipah virus |  | P |  |  | P |  | L |  |  | P |  |  |
| Rabies viruses* | P | P | P | P | P | P | P |  | P | P | P | P |
| Rift Valley fever virus | P | P | P |  | P |  |  |  |  | P |  |  |
| Rotavirus B |  |  | L |  |  |  | L |  |  |  |  |  |
| SARS virus (or SARS-like CoV) |  | P | P | P | P |  | P |  |  | P |  |  |
| South American hemorrhagic fever arenaviruses* |  |  | P |  |  |  |  |  |  |  |  |  |
| **Viral diseases (vector-borne)** |  |  |  |  |  |  |  |  |  |  |  |  |
| California encephalitis |  | L |  |  |  | L | L |  | L |  |  |  |
| Chikungunya virus |  | L | L |  |  |  |  |  |  |  |  |  |
| Dengue virus | L | L |  |  |  |  |  |  |  |  |  |  |
| Eastern equine encephalitis virus |  | L |  |  |  | L |  |  |  |  |  |  |
| Japanese encephalitis virus |  | L |  |  | L |  | L |  |  |  |  |  |
| St. Louis encephalitis virus | L | L | L |  | L | L |  |  | L |  |  |  |
| Tick-borne encephalitis virus complex* | P | P | P |  | P |  |  |  | P |  |  |  |
| Venezuelan equine encephalitis virus |  | L |  |  |  | L | P |  | L |  |  |  |
| West Nile virus | L | L |  |  |  | L | L |  |  |  |  |  |
| Western equine encephalitis virus | L |  | L |  |  |  |  |  | L |  |  |  |
| Yellow fever virus |  | L |  |  |  |  |  |  |  |  |  |  |
| **Parasitic diseases** |  |  |  |  |  |  |  |  |  |  |  |  |
| *Echinicoccus spp* | P |  | P |  |  | P | P |  | P | P |  |  |
| **Bacterial diseases** |  |  |  |  |  |  |  |  |  |  |  |  |
| *Bacillus anthracis* |  |  | P | P |  | P | P |  |  | P |  | P |
| *Brucella spp.* |  |  | P |  |  | P | P |  | P | P |  |  |
| *Coxiella burnetii* | P |  | P |  |  | P |  |  | P | P |  |  |
| *Francisella tularensis* | P |  | P |  |  | P |  |  | P | P |  |  |
| *Leptospira spp.* | P |  | P | P |  | P | P |  | P | P |  |  |
| *Mycobacterium tuberculosis* complex* |  |  | P | P |  | P | P | P | P | P | P |  |
| *Yersinia pestis* | P |  | P |  |  | P |  |  | P | P |  |  |
| **Total potential Significant Zoonoses (i.e., Significant Zoonosis found within family)** | **15** | **17** | **26** | **7** | **9** | **15** | **18** | **1** | **15** | **14** | **3** | **3** |

*Rabies viruses included the zoonotic lyssaviruses Australian bat lyssavirus, Duvenhage, European bat lyssavirus 1 and 2, Mokolo, and rabies; tick-borne encephalitis complex included Kyasanur Forest disease, Omsk hemorrhagic fever, and tickborne encephalitis; Ebolaviruses included Bundibugyo, Côte d’Ivoire, Reston, Sudan and Zaire; South American hemorrhagic fever arenaviruses included Guanarito, Junin, Machupo, and Sabia; hantaviruses associated with HFRS included Dobrava, Hantaan, Puumala, Saaremaa, and Seoul; hantaviruses associated with HCPS included Andes, Bayou, Black Creek Canal, Laguna Negra, New York, and Sin Nombre; *Mycobacterium tuberculosis* complex species included *M. africanum, M. bovis, M. bovis BCG, M. caprae, M. microti, M. pinnipedii,* and *M. tuberculosis homini* [24].
