# Supplementary material for: Wildlife Trade and Human Health in Lao PDR: An Assessment of the Zoonotic Disease Risk in Markets
Source: PLoS One. 2016 Mar 23;11(3):e0150666. doi: 10.1371/journal.pone.0150666 (PMC4805265; doi:10.1371/journal.pone.0150666)
Supplement: S2 Table — Species included are those classified as Category I, species that are rare or near extinct or Category II, species where if management is neglected, they will become extinct. (DOCX) [file pone.0150666.s006.docx]

**S2 Table**. **Species observed being traded that are listed in the Lao PDR Wildlife and Aquatic Law.** Species included are those classified as Category I, species that are rare or near extinct or Category II, species where if management is neglected, they will become extinct.

|  | **Wildlife and Aquatic Law Category** | |
| --- | --- | --- |
| **Species** | **Category I** | **Category II** |
| *Pardofelis marmorata* | 1 |  |
| *Elephas maximus* | 3 |  |
| *Ophiophagus hannah* | 3 |  |
| *Prionodon pardicolor* | 3 |  |
| *Platysternon megacephalum* | 4 |  |
| *Viverricula indica* | 6 |  |
| *Nycticebus pygmaeus* | 8 |  |
| *Python reticulatus* | 8 |  |
| *Lophura diardi* | 11 |  |
| *Bos gaurus* | 14 |  |
| *Laonastes aenigmamus* | 18 |  |
| *Manouria impressa* | 19 |  |
| *Centropus sinensis* | 20 |  |
| *Cervus unicolor* | 22 |  |
| *Polyplectron bicalcaratum* | 33 |  |
| *Nycticebus bengalensis* | 45 |  |
| *Lophura nycthemera* | 136 |  |
| *Ardea purpurea* |  | 1 |
| *Athene brama* |  | 1 |
| *Eonycteris spelaea* |  | 1 |
| *Glaucidium brodiei* |  | 1 |
| *Heosemys grandis* |  | 1 |
| *Hieremys annandalii* |  | 1 |
| *Muntiacus muntjak* |  | 1 |
| *Ninox scutulata* |  | 1 |
| *Pyxidea mouhotii* |  | 1 |
| *Rhizomys pruinosus* |  | 1 |
| *Treron bicincta* |  | 1 |
| *Viverra tangalunga* |  | 1 |
| *Strix leptogrammica* |  | 2 |
| *Treron curvirostra* |  | 2 |
| *Tyto alba* |  | 2 |
| *Viverra megaspila* |  | 2 |
| *Acridotheres tristis* |  | 3 |
| *Alcedo atthis* |  | 3 |
| *Amyda cartilaginea* |  | 3 |
| *Egretta garzetta* |  | 3 |
| *Herpestes javanicus* |  | 3 |
| *Hipposideros armiger* |  | 3 |
| *Treron apicauda* |  | 3 |
| *Treron sieboldii* |  | 3 |
| *Bubulcus ibis* |  | 4 |
| *Cuora amboinensis* |  | 4 |
| *Indotestudo elongata* |  | 4 |
| *Tragulus kanchil* |  | 4 |
| *Arctictis binturong* |  | 5 |
| *Melogale moschata* |  | 7 |
| *Varanus salvator* |  | 9 |
| *Anthracoceros albirostris* |  | 13 |
| *Glaucidium cuculoides* |  | 18 |
| *Petaurista philippensis* |  | 19 |
| *Streptopelia tranquebarica* |  | 19 |
| *Paguma larvata* |  | 22 |
| *Ratufa bicolor* |  | 29 |
| *Columba punicea* |  | 31 |
| *Gracula religiosa* |  | 31 |
| *Hystrix brachyuran* |  | 35 |
| *Malayemys subtrijuga* |  | 45 |
| *Lepus peguensis* |  | 52 |
| *Atherurus macrourus* |  | 69 |
| *Rhizomys sumatrensis* |  | 71 |
| *Physignathus cocincinus* |  | 114 |
| *Hipposideros larvatus* |  | 126 |
| *Paradoxurus hermaphrodites* |  | 136 |
| *Tupaia belangeri* |  | 156 |
| *Ptyas mucous* |  | 204 |
| *Spilopelia chinensis* |  | 280 |
| *Varanus bengalensis* |  | 357 |
| Unidentified *Chiroptera** |  | 3411 |
| Unidentified *Bucerotidae** | 1 |  |
| Unidentified *Ursidae** | 4 |  |
| Unidentified *Naemorhedus** | 11 |  |
| Unidentified *Capricornis** | 12 |  |
| Unidentified *Pitta** |  | 1 |
| Unidentified *Herpestidae** |  | 5 |
| Unidentified *Strigidae** |  | 15 |
| Unidentified *Ardeidae** |  | 45 |
| Unidentified *Muntiacus** |  | 108 |
| Unidentified *Tragulus** |  | 160 |
| Unidentified *Psittacula** |  | 195 |
| Unidentified *Treron** |  | 222 |
| **Total** | **382** | **6070** |

*Although species was not identified, all species from genus/family/order, found in Lao PDR, are included in Category I or Category II of the Wildlife and Aquatic Law.
