# Supplementary material for: Wildlife Trade and Human Health in Lao PDR: An Assessment of the Zoonotic Disease Risk in Markets
Source: PLoS One. 2016 Mar 23;11(3):e0150666. doi: 10.1371/journal.pone.0150666 (PMC4805265; doi:10.1371/journal.pone.0150666)
Supplement: S3 Table — Species included as those classified as Critically Endangered (CR), Endangered (EN), Vulnerable (V) or Near Threatened (NT). (DOCX) [file pone.0150666.s007.docx]

**S3 Table**. **Species observed being traded that are listed on the IUCN Redlist.** Species included as those classified as Critically Endangered (CR), Endangered (EN), Vulnerable (V) or Near Threatened (NT).

|  | **IUCN Redlist category** | | | |
| --- | --- | --- | --- | --- |
| **Species** | **CR** | **EN** | **VU** | **NT** |
| *Mauremys annamensis* | 1 |  |  |  |
| *Hieremys annandalii* |  | 1 |  |  |
| *Pyxidea mouhotii* |  | 1 |  |  |
| *Elephas maximus* |  | 3 |  |  |
| *Indotestudo elongata* |  | 4 |  |  |
| *Platysternon megacephalum* |  | 4 |  |  |
| *Laonastes aenigmamus* |  | 18 |  |  |
| *Heosemys grandis* |  |  | 1 |  |
| *Macaca leonina* |  |  | 1 |  |
| *Pardofelis marmorata* |  |  | 1 |  |
| *Lonchura oryzivora* |  |  | 2 |  |
| *Viverra megaspila* |  |  | 2 |  |
| *Amyda cartilaginea* |  |  | 3 |  |
| *Ophiophagus hannah* |  |  | 3 |  |
| *Cuora amboinensis* |  |  | 4 |  |
| *Arctictis binturong* |  |  | 5 |  |
| *Nycticebus pygmaeus* |  |  | 8 |  |
| *Bos gaurus* |  |  | 14 |  |
| *Manouria impressa* |  |  | 19 |  |
| *Cervus unicolor* |  |  | 22 |  |
| *Columba punicea* |  |  | 31 |  |
| *Malayemys subtrijuga* |  |  | 45 |  |
| *Nycticebus bengalensis* |  |  | 45 |  |
| *Terpsiphone atrocaudata* |  |  |  | 1 |
| *Phaenicophaeus diardi* |  |  |  | 2 |
| *Psittacula alexandri* |  |  |  | 2 |
| *Viverra zibetha* |  |  |  | 2 |
| *Dicrurus andamanensis* |  |  |  | 5 |
| *Cyclemys dentata* |  |  |  | 7 |
| *Ratufa bicolor* |  |  |  | 29 |
| **Total** | **1** | **31** | **206** | **48** |
